# Diabetes abrogates sex differences and aggravates cardiometabolic risk in postmenopausal women

**DOI:** 10.1186/1475-2840-12-61

**Published:** 2013-04-09

**Authors:** Filipa Mascarenhas-Melo, Daniela Marado, Filipe Palavra, José Sereno, Álvaro Coelho, Rui Pinto, Edite Teixeira-Lemos, Frederico Teixeira, Flávio Reis

**Affiliations:** 1 Laboratory of Pharmacology & Experimental Therapeutics, IBILI, Faculty of Medicine, University of Coimbra, Azinhaga de Santa Comba, Celas, Coimbra, 3000-548, Portugal; 2 Internal Medicine Department, General Hospital, University and Hospital Centre of Coimbra, Quinta dos Vales, S. Martinho do Bispo, Coimbra, 3041-801, Portugal; 3 Neurology Department, General Hospital, University and Hospital Centre of Coimbra, Quinta dos Vales, S. Martinho do Bispo, Coimbra, 3041-801, Portugal; 4 Pharmacology and Pharmacotoxicology Unit, Faculty of Pharmacy, University of Lisbon, Av. Prof. Gama Pinto, Lisbon, 1649-003, Portugal; 5 ESAV, Polytechnic Institute of Viseu, Av. Cor. José Maria Vale de Andrade, Campus Politécnico, Viseu, 3504-510, Portugal

**Keywords:** Diabetes, Gender, HDL-c subpopulations, Markers of cardiovascular risk, Menopause

## Abstract

**Background:**

The aim of this study is to evaluate the effect of gender and menopause in cardiometabolic risk in a type 2 diabetes mellitus (T2DM) population, based on classical and non-traditional markers.

**Methods:**

Seventy four volunteers and 110 T2DM patients were enrolled in the study. Anthropometric data, blood pressure, body mass index (BMI), waist circumference (WC) and the following serum markers were analyzed: glucose, Total-c, TGs, LDL-c, Oxidized-LDL, total HDL-c and large and small HDL-c subpopulations, paraoxonase 1 activity, hsCRP, uric acid, TNF-α, adiponectin and VEGF.

**Results:**

Non-diabetic women, compared to men, presented lower glycemia, WC, small HDL-c, uric acid, TNF-α and increased large HDL-c. Diabetes abrogates the protective effect of female gender, since diabetic women showed increased BMI, WC, small HDL-c, VEGF, uric acid, TNF-α and hsCRP, as well as reduced adiponectin, when compared with non-diabetic. In diabetic females, but not in males, WC is directly and significantly associated with TNF-α, VEGF, hsCRP and uric acid; TNF-α is directly associated with VEGF and hsCRP, and inversely with adiponectin. Postmenopausal females presented a worsen cardiometabolic profile, viewed by the increased WC, small HDL-c, VEGF, uric acid, TNF-α and hsCRP. In this population, WC is directly and significantly associated with TNF-α, VEGF, hsCRP; TNF-α is directly associated with VEGF; and uric acid is inversely associated with large HDL-c and hsCRP with adiponectin, also inversely.

**Conclusions:**

Diabetes abrogates the protective effect of gender on non-diabetic women, and postmenopausal diabetic females presented worsen cardiometabolic risk, including a more atherogenic lipid sketch and a pro-inflammatory and pro-angiogenic profile. The classical cardiovascular risk factors (CVRFs) fail to completely explain these differences, which are better clarified using “non-traditional” factors, such as HDL-c subpopulations, rather than total HDL-c content, and markers of inflammation and angiogenesis, namely TNF-α, hsCRP, uric acid and VEGF. Multi-therapeutic intervention, directed to obesity, atherogenic lipid particles and inflammatory mediators is advisory in order to efficiently prevent the serious diabetic cardiovascular complications.

## Background

Cardiovascular disease (CVD) is a major public health problem worldwide and the leading cause of death in Portugal and in most industrialized and developing countries [1,2]. This mortality of cardiovascular (CV) cause appears to be increasing in countries where type 2 diabetes mellitus (T2DM) is prevalent; therefore, diabetic patients are associated with a risk 2 to 4 times higher of CVD development, accounting for 50-80% of deaths as well as for the increased morbidity and loss of quality of life in these patients [3,4]. Apart from being per se a risk factor for CVD, T2DM is often associated with a higher prevalence of other important risk factors, including hypertension, obesity, insulin resistance, microalbuminuria and dyslipidaemia [5,6], a multifactorial condition, often referred as metabolic syndrome, which is responsible for the high CV morbidity and mortality in those patients [7]. Pharmacological treatment is crucial for delaying the progression of diabetes, yet it remains inadequate in preventing the increased risk of CVD in patients with T2DM, especially women [6].

The CV risk seems to be different for men and women. The apparent cardioprotective effects of endogenous estrogens seem to prevent CVD in premenopausal women, when compared with age-matched men; however, following menopause and the consequent loss of hormonal effects, gender-based differences in CVD are reduced [8,9]. The increased CVD risk after menopause seems to be associated with the emergence of the features of metabolic syndrome [10], but the precise causes remain to be fully elucidated. Obesity is an independent risk factor for macrovascular disease across sexes [11]; however, despite higher incidence of obesity in premenopausal women, rates of macrovascular disease are lower than in men. Interestingly, this sex difference, which normally vanishes after menopause, is rapidly lost in premenopausal T2DM patients, with CVD reaching 2- to 5-fold higher rates than in non-diabetic women [12]. In fact, women with T2DM, compared with age-matched non-diabetic women, exhibit several-fold higher rates of death related to coronary artery disease (CAD), with event rates nearly identical to those observed in T2DM men [13]. Traditional cardiovascular risk factors (CVRFs) cannot completely account for these sex differences in cardiovascular mortality [14]. So, it seems clear that more studies are needed to understand the precise influence of gender and menopause in the risk for CVD, especially in diabetic patients, in order to achieve effective preventative and disease management strategies to reduce the CVD risk associated with the disease, particularly in postmenopausal women.

As the leading cause of death in T2DM patients, the CVD is a complex phenomenon, which involves metabolic, lipidic, oxidative, inflammatory, as well as genetic factors. Beyond hyperglycemia, a number of other common risk factors may contribute to CVD in diabetic women. Low plasma levels of high-density lipoprotein cholesterol (HDL-c) have been largely recognized as a risk factor for coronary heart disease (CHD) [15,16] and they are a common feature of the dyslipidaemia linked to diabetes and insulin resistance [17]. Recent findings suggest that monitoring the type of HDL particles (carry distinct and specific proteins or lipids and differentiated by their density and size – large, intermediate and small), rather than their total quantity, is a more reasonable way of determining the CV risk, suggesting that different subpopulations may have a different role in reverse cholesterol transport and CVD risk protection [18]. In fact, some recent studies have been reporting that large HDL levels are reduced in patients with CAD compared to healthy subjects and inversely related to both disease severity and progression of coronary lesions [19]. Paraoxonases (PONs) are enzymes from the HDL, and have been indicated as one of the best candidates for the protective activity of HDL against CVD development, namely protection against LDL oxidation [20]. In fact, oxidized LDL (Ox-LDL) and HDL are indeed antagonists in the development of CVD [21]. Several studies have shown a strong positive correlation between the lower levels of HDL and the development of atherosclerosis, but their role in the determination of CVR in gender and menopause in a diabetic population remains to be clarified.

Chronic inflammation is currently viewed as a key factor in the development of atherosclerosis, contributing to raise the overall CV risk, namely in diabetic patients. An inflammatory imbalance, as manifested by increased pro-inflammatory cytokines, such as the tumor necrosis factor alpha (TNF-α), increased C-reactive protein (hsCRP), and/or reduced levels of anti-inflammatory and anti-atherogenic mediators, such as adiponectin, have been considered a key factor for the increased CVR in some pathologies [22,23], deserving more attention in respect to the gender and menopause, mainly in diabetes. Similar importance is now attributed to the phenomenon of angiogenesis, which has the vascular endothelial growth factor (VEGF) as the key biomarker, which has been suggested as a novel promising target for T2DM treatment [24]. Another new marker that deserves our attention is the uric acid; although uric acid can act as an antioxidant, excess serum accumulation is often associated with several conditions, and has been suggested as an independent risk factor for carotid atherosclerosis in patients with type 2 diabetes [25]. Furthermore, a prospective follow-up study showed that hyperuricemia is associated with higher risk of T2DM, independent of obesity, dyslipidemia and hypertension [26]. Thus, it would be important to understand its influence concerning gender and menopause modulation of CV risk, mainly in diabetes.

This study aimed to evaluate the influence of gender and menopause on CV risk in a diabetic population, using both traditional and new non-traditional markers.

## Materials and methods

### Subjects and ethical consideration

As control group 74 volunteers, including 39 males and 35 females (16 pre- and 19 postmenopausal), were randomly recruited during the performance of routine laboratory analysis in a clinical laboratory. Participants were not using exogenous steroids, not taking any medication and did not declare any disease. The study involved also 110 age and gender-matched T2DM patients, including 57 males and 53 females (8 pre- and 45 postmenopausal). Type 2 diabetes mellitus was diagnosed in the Diabetes and Metabolic Diseases Unit from the Coimbra Hospital Centre (EPE), according to the European Guidelines. Diabetes patients were treated with the following medication: a) Insulin and/or oral antidiabetic drugs (OAD): biguanides monotherapy (14), sulfonylurea monotherapy (3), combination of two OAD (25), combination of three or more OAD (32), combination of OAD and insulin (20), insulin monotherapy (16); b) antidyslipidemic drugs: statins monotherapy (39), fibrates monotherapy (4), combination of two antidyslipidemic drugs(7) and without antidyslipidemic drugs (60); c) antihypertensive drugs, mainly angiotensin-converting enzyme inhibitors, angiotensin receptor blockers, calcium channel blockers and diuretics, distributed by the following protocols: monotherapy (18), combination of two antihypertensive drugs (34), combination of three or more antihypertensive drugs (35) and without antihypertensive drugs (23). Menopausal status (pre- and postmenopausal women) was defined by the questionnaire and menstrual status was self-reported during the interviews. Pregnant women and people with age <16 or >75 years were excluded from this study. The study was performed in agreement with the code of ethics of the World Medical Association (Declaration of Helsinki) for human studies and received authorization from the local ethics committee, as well as from all the participants by signing a written informed consent.

### Data and blood collection

The following data was obtained from each subject by trained personnel: weight and height (without shoes and wearing light outdoor clothing) were measured in order to calculate body mass index (BMI), waist circumference (WC), as well as systolic and diastolic blood pressure (SBP and DBP), the latter of which were assessed in the sitting position after a 5-min rest. Blood samples were collected by venipuncture from the subjects after an overnight fasting period, via both EDTA-containing tubes and tubes without anticoagulant, in order to obtain plasma, buffy-coat and serum, and processed within 2 hours of collection. Aliquots were immediately stored at −80°C until assayed.

### Assays

#### Lipid profile

Serum total cholesterol (Total-c), HDL cholesterol (HDL-c), LDL cholesterol (LDL-c) and triglycerides (TGs) were analyzed on a Hitachi 717 analyser (Roche Diagnostics) using standard laboratorial methods. Total-c reagents and TGs kit were obtained from bioMérieux® sa (Lyon, France). HDL-c Plus and LDL-c Plus tests were obtained from F. Hoffmann-La Roche Ltd (Roche Diagnostics Div., Basel, Switzerland). Serum glucose levels were measured using a Glucose Oxidase commercial kit (Sigma, St. Louis, Mo, USA). Plasma concentration of Ox-LDL was evaluated by using a standard commercial enzyme-linked immunoassay (Oxidized LDL ELISA, Mercodia, Uppsala, Sweden).

#### HDL subpopulations assay

Subpopulations were separated and quantified using a Lipoprint kit from Quantimetrix Corp. (Redondo Beach, CA, USA). The assay involves a polyacrylamide gel electrophoresis assay and a complete Lipoprint System for data acquisition and quantification of Large, Intermediate, and Small subpopulations of HDL.

#### PON1 paraoxonase activity

Was assessed spectrophotometrically and expressed in nmol of pnitrophenol/ml/min. In brief, paraoxonase activity was measured by adding serum to 1 mL Tris/HCl buffer (100 mmol/L, pH 8.0) containing 2 mmol/L CaCl2 and 5.5 mmol/L paraoxon (O,O-diethyl-O-p-nitrophenylphosphate; Sigma Chemical Co). The rate of generation of p-nitrophenol was determined at 412 nm, 37°C, via the use of a continuously recording spectrophotometer (Beckman DU-68).

#### Serum inflammatory, angiogenic and endothelial markers

Serum adiponectin, TNF-α and VEGF contents were assessed using Quantikine® enzyme-linked immunoassays kits from R&D Systems (Minneapolis, USA); serum intercellular adhesion molecule 1 (iCAM1) levels were evaluated by using an Elisa kit from Abcam (Cambridge, MA, USA); high-sensitivity C-reactive protein (hsCRP) was evaluated by immunoturbidimetry, using commercially available kits (CRP [latex] High-Sensitivity, Roche Diagnostics); uric acid was analyzed on a Hitachi 717 analyser (Roche Diagnostics) using standard laboratory methods.

### Statistical analysis

Statistical analysis was performed by using the IBM Statistical Package for Social Sciences (SPSS) for Windows, version 20.0, (SPSS Inc., Chicago, IL, USA). The distribution of continuous variables was analyzed using Kolmogorov-Smirnov tests, to assess significant departures from Normality. Comparisons between groups were performed using the Independent Samples *t*-test and the Mann–Whitney test. Adjustment of statistical differences for confounding factors was performed using analysis of covariance (ANCOVA). The association between categorical variables was analyzed using Pearson’s test. Statistical significance was accepted at P less than 0.05.

## Results

### Anthropometric data and general characterization of populations

**Figure 1:**
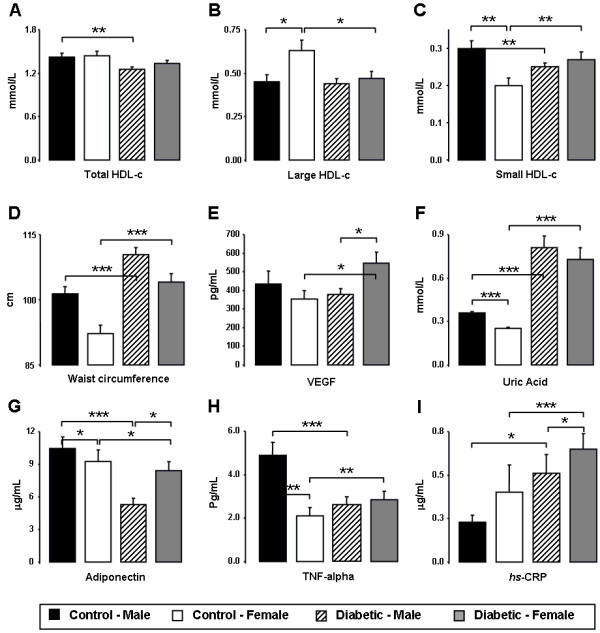
**Gender effect on control and diabetic populations.** Serum total HDL-c (**A**), large HDL-c (**B**), small HDL-c (**C**), waist circumference (**D**), VEGF (**E**), uric acid (**F**), adiponectin (**G**), TNF-α (**H**) and *hsCRP* (**I**), in male and female diabetic patients and controls. Results are presented as mean ± SEM. *p<0.05, **p<0.01 and ***p<0.001.

The demographic and anthropometric data of control volunteers and diabetic patients (male, female, pre- and postmenopausal women) are summarized in Tables 1 and 2. Seventy four control volunteers were enrolled in the study: 39 (52.70%) males and 35 (47.30%) females (16 in the premenopausal and 19 in the postmenopausal stage). One hundred and ten type 2 diabetic patients were recruited: 57 (51.82%) males and 53 (48.18%) females (8 in the premenopausal and 45 in the postmenopausal stage). Diabetic patients, both male and female, presented significantly higher values of glycemia, BMI, WC and uric acid when compared with the gender and age-matched controls (Table 1 and Figure 1D). Systolic and diastolic blood pressure was significantly lower in the male and female diabetic subjects, which is in agreement with the antihypertensive medication taken. Regarding differences between males and females in each population, the female controls presented significantly reduced values of glycemia and SBP, when compared with the age-matched control males, while the female diabetic patients presented significantly higher of glycated hemoglobin (HbA1c), when compared with the age-matched diabetic males (Table 1). BMI and WC values should not be compared between different sexes as the normal range values are distinct for man and woman.

**Table 1:** Data from the diabetic population and age and gender-matched controls

		**Control group**			**Diabetic group**		**Diabetic vs control (P)**	
**Parameters**	**Male (n = 39)**	**Female (n = 35)**	**P**	**Male (n = 57)**	**Female (n = 53)**	**P**	**Male**	**Female**
Age (years)	59.97 ± 1.02	54.71 ± 1.68	0.100	58.88 ± 1.34	60.49 ± 1.42	0.524	0.453	0.013
Gender (%M/%F)	52.70	47.30	—	51.82	48.18	—	—	—
BMI (Kg/m^2^)	27.89 ± 0.72	27.09 ± 0.71	0.704	30.37 ± 0.65	30.12 ± 0.67	0.788	0.013	0.002
WC (cm)	101.32 ± 1.75	92.26 ± 1.96	0.003	110.36 ± 1.70	104.02 ± 2.05	0.020	0.000	0.000
SBP (mmHg)	145.28 ± 3.08	143.74 ± 3.98	0.010	136.86 ± 2.95	137.53 ± 3.61	0.942	0.002	0.000
DBP (mmHg)	87.10 ± 1.54	87.76 ± 2.06	0.654	73.54 ± 1.69	72.74 ± 1.77	0.745	0.000	0.000
Glycemia (mmol/L)	5.58 ± 0.08	5.12 ± 0.09	0.000	9.70 ± 0.48	10.69 ± 0.59	0.191	0.000	0.000
HbA1c (%)	—	—	—	8.13 ± 0.25	9.20 ± 0.30	0.007	—	—
Total-c (mmol/L)	5.71 ± 0.15	5.30 ± 0.14	0.121	4.75 ± 0.15	4.89 ± 0.15	0.526	0.000	0.129
TGs (mmol/L)	1.28 ± 0.08	1.08 ± 0.06	0.118	1.85 ± 0.15	1.99 ± 0.15	0.406	0.002	0.000
LDL-c (mmol/L)	3.70 ± 0.15	3.36 ± 0.13	0.222	2.69 ± 0.14	2.69 ± 0.14	0.996	0.000	0.012
Ox-LDL (U/L)	52.65 ± 2.60	33.23 ± 1.92	0.000	32.58 ± 1.78	32.42 ± 1.84	1.000	0.000	0.401
Non-HDL-c (mmol/L)	4.28 ± 0.15	3.86 ± 0.14	0.110	3.50 ± 0.16	3.56 ± 0.16	0.635	0.004	0.257
PON1 activity	519.66 ± 18.63	449.05 ± 21.66	0.034	426.01 ± 20.27	490.17 ± 30.31	0.077	0.004	0.780

Results are presented as media ± SEM. Independent samples *T*-test and Mann–Whitney test for normalized and non-normalized samples, respectively. P adjusted for age and BMI (ANCOVA) when adequate (when p<0.05): for age when comparing female diabetic vs female control subjects; for BMI when comparing diabetic vs control (both for male and for female). BMI, body mass index; HbA1c, glycated hemoglobin; SBP, systolic blood pressure; DBP, diastolic blood pressure; LDL-c, low-density lipoprotein cholesterol; Ox-LDL, oxidized low-density lipoprotein; TGs, triglycerides; Total-c, total cholesterol; WC, waist circumference.

**Table 2:** Data from the female diabetic population and age-matched controls: menopause influence

	**Female control group**			**Female diabetic group**			**Diabetic vs control (P)**	
**Parameters**	**Pre-M (n = 16)**	**Post-M (n = 19)**	**P**	**Pre-M (n = 8)**	**Post-M (n = 45)**	**P**	**Pre-menopausal**	**Post- menopausal**
Age (years)	45.63 ± 1.12	62.37 ± 1.35	0.000	43.88 ± 1.29	63.44 ± 1.20	0.000	0.349	0.345
BMI (Kg/m^2^)	26.93 ± 1.21	27.21 ± 0.85	0.472	29.43 ± 2.44	30.24 ± 0.67	0.578	0.221	0.013
WC (cm)	91.38 ± 2.86	93.06 ± 2.75	0.575	100.57 ± 4.93	104.66 ± 2.27	0.081	0.103	0.000
SBP (mmHg)	132.69 ± 5.62	153.56 ± 4.61	0.004	120.38 ± 7.22	140.58 ± 3.91	0.048	0.187	0.113
DBP (mmHg)	84.25 ± 3.20	90.89 ± 2.52	0.175	72.25 ± 3.14	72.82 ± 2.02	0.519	0.027	0.000
Glycemia (mmol/L)	4.92 ± 0.10	5.31 ± 0.14	0.038	10.80 ± 1.65	10.67 ± 0.64	0.832	0.000	0.000
HbA1c (%)	—	—	—	9.03 ± 1.08	9.23 ± 0.30	0.477	—	—
Total-c (mmol/L)	5.08 ± 0.16	5.48 ± 0.21	0.364	4.68 ± 0.44	4.93 ± 0.16	0.725	0.313	0.090
TGs (mmol/L)	1.02 ± 0.08	1.13 ± 0.10	0.455	1.51 ± 0.31	2.08 ± 0.17	0.467	0.066	0.003
LDL-c (mmol/L)	3.21 ± 0.17	3.47 ± 0.19	0.514	2.71 ± 0.35	2.69 ± 0.15	0.997	0.164	0.007
Ox-LDL (U/L)	29.13 ± 1.76	36.46 ± 2.98	0.044	34.83 ± .58	31.89 ± 1.61	0.993	0.897	0.339
Non-HDL-c (mmol/L)	3.68 ± 0.17	4.01 ± 0.20	0.390	3.36 ± 0.45	3.60 ± 0.17	0.781	0.236	0.243
PON1 activity	415.66 ± 26.78	477.17± 32.14	0.420	443.94 ± 31.40	498.39 ± 35.22	0.089	0.527	0.916

Results are presented as media ± SEM. Independent samples *T*-test and Mann–Whitney test for normalized and non-normalized samples, respectively. P adjusted for age and BMI (ANCOVA) when adequate (when p<0.05): for age when comparing pre vs postmenopausal women (both in control and diabetic subjects); for BMI when comparing postmenopausal diabetic women vs postmenopausal control subjects. BMI, body mass index; HbA1c, glycated hemoglobin; SBP, systolic blood pressure; DBP, diastolic blood pressure; LDL-c, low-density lipoprotein cholesterol; Ox-LDL, oxidized low-density lipoprotein; TGs, triglycerides; Total-c, total cholesterol; WC, waist circumference.

**Figure 2:**
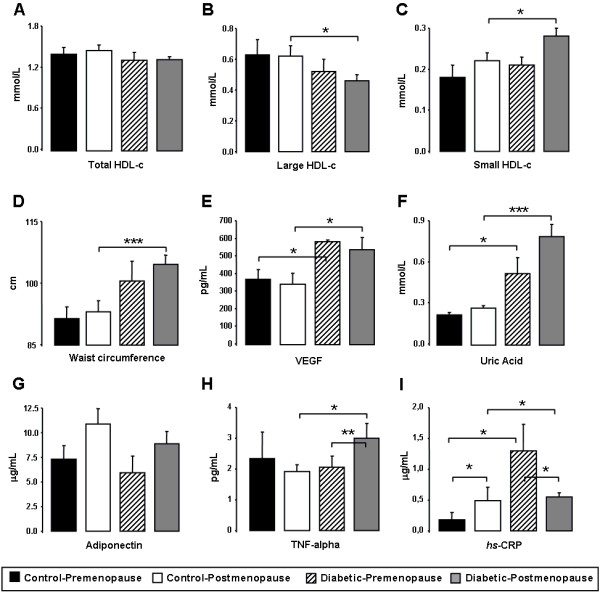
**Menopause effect on control and diabetic populations.** Serum total HDL-c (**A**), large HDL-c (**B**), small HDL-c (**C**), waist circumference (**D**), VEGF (**E**), uric acid (**F**), adiponectin (**G**), TNF-α (**H**) and *hsCRP* (**I**), in pre and postmenopausal diabetic patients and controls. Results are presented as mean ± SEM. *p<0.05, **p<0.01 and ***p<0.001.

Concerning the pre and postmenopausal populations of controls and diabetics females, premenopausal diabetic patients presented significantly higher values of glycemia and a trend (although non-statistically significant) to increased BMI and WC, when compared with the premenopausal control women. Postmenopausal diabetic women presented additional changes. Indeed, when compared with the age-matched postmenopausal control females, diabetic presented significantly increased glycemia, BMI and WC (Table 2 and Figure 2D). Diastolic blood pressure was significantly lower in the diabetic females (both pre and postmenopausal) vs the control ones. Concerning differences before and after menopause in each population, postmenopausal control women showed increased glycemia and SBP versus premenopausal, while postmenopausal diabetic females presented increased SBP without changes on BMI or WC when compared with premenopausal diabetic patients (Table 2 and Figure 2D). Values were analyzed after age and BMI adjustment when adequate.

### Classical lipid profile

As previously mentioned, diabetic patients were under antidyslipidemic therapy, which justify some of the data obtained for the classic lipid profile. Therefore, male diabetic patients presented significantly lower serum concentrations of Total-c, LDL-c, Ox-LDL-c and non-HDL-c when compared with the male control subjects. Identical profile was encountered between female diabetic and control individuals, with significantly lower serum contents of LDL-c and a trend to reduced Total-c, Ox-LDL-c and non-HDL-c for the female diabetic women (Table 1). However, TGs and HDL-c concentrations showed a distinct profile, most probably due to the expected lower impact of antidyslipidemic medication on these parameters of lipid profile. In agreement, male diabetic patients presented significantly higher values of TGs and lower of HDL-c, when compared with controls, and female patients presented also increased TGs contents and a trend to reduced HDL-c, when compared with female controls (Figure 1A). Regarding differences between males and females in each population, only a reduced Ox-LDL-c concentration was found in the females of the control group (vs males), without changes between females and males in the diabetic population (Table 1).

Concerning the pre and postmenopausal populations of control and diabetic females, premenopausal patients presented significantly higher values of TGs when compared with premenopausal controls, while postmenopausal diabetic showed reduced LDL-c and increased TGs (Table 2), without changes on the other classical lipid profile parameters, vs controls. Regarding differences between pre and postmenopausal women in each population, only an increased Ox-LDL-c concentration was found in the postmenopausal control women (vs premenopausal), without changes between pre and postmenopausal diabetic women (Table 2).

### Paraoxonase activity and HDL-c subpopulations

Unchanged values of paraoxonase activity were found between diabetic and control women, while a reduced activity was encountered in male diabetic patients when compared with the male control subjects. Regarding differences within the groups, female controls presented significantly lower values of PON1 activity (vs male), without changes between male and female diabetic patients (Table 1). No changes were encountered between pre and postmenopausal women in the control and in the diabetic populations, as well as between the diabetic and matched controls (before or after menopause).

In relation to HDL-c subpopulations, diabetic women presented significantly lower concentration of large HDL-c and higher of small HDL-c, when compared with female controls; male patients presented unchanged values of large HDL-c and significantly lower of small HDL-c, when compared with male controls. Furthermore, while unchanged values were found between diabetic male and female for both large and small HLD-c contents, increased large and reduced small HDL-c concentrations were found in the female controls when compared with males (Figure 1B and 1C).

In respect to menopause influences, unchanged values were found between pre and postmenopausal status for large and small HDL in the control population, as well as in the diabetic patients. However, postmenopausal diabetic women presented significantly reduced large HDL-c and increased small HDL-c concentrations, when compared with postmenopausal controls, while unchanged values for both large and small HDL-c subpopulations were encountered between premenopausal diabetic patients and controls (Figure 2B and 2C).

### Markers of inflammation, angiogenesis and endothelial lesion

The diabetic women presented increased levels of serum VEGF, uric acid, hsCRP and TNF-α and reduced of adiponectin, when compared with the control females, while male diabetic patients showed increased serum uric acid and hsCRP concentrations and reduced TNF-α and adiponectin, when compared with male control subjects (Figure 1E, 1F, 1G, 1H and 1I). Regarding differences between male and female, diabetic women presented significantly increased levels of serum VEGF, hsCRP and adiponectin (versus male patients), while control females presented significantly reduced values of uric acid, TNF-α and adiponectin, when compared with male controls (Figure 1F, 1G, 1H and 1I).

Concerning menopause influence, postmenopausal diabetic patients presented significantly increased serum uric acid, hsCRP, TNF-α and VEGF contents, and unchanged of adiponectin, when compared with the postmenopausal controls. Premenopausal patients presented significantly higher uric acid, hsCRP and VEGF concentrations, and unchanged of TNF-α and adiponectin, versus the premenopausal controls (Figure 2E, 2F, 2G, 2H and 2I). Regarding differences before and after menopause, control postmenopausal subjects only presented increased serum hsCRP contents, versus premenopausal control women, while diabetic postmenopausal patients showed increased TNF-α and reduced hsCRP contents (Figure 2E, 2F, 2G, 2H and 2I).

### Analysis of correlations between markers of CV risk in diabetic patients

**Figure 3:**
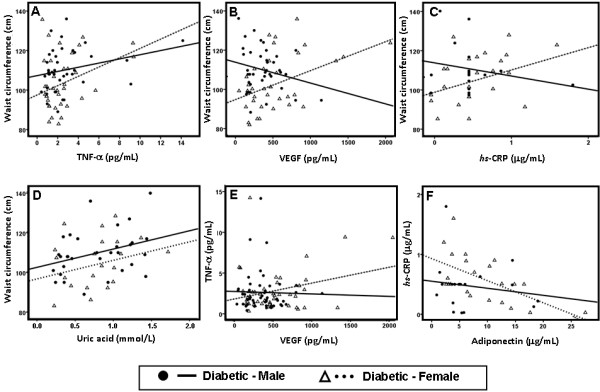
**Main correlations in male and female diabetic patients.** Correlation between waist circumference and TNF-α (**A**), VEGF (**B**), *hsCRP* (**C**) and uric acid (**D**); between TNF-α and VEGF (**E**) and between *hsCRP* and adiponectin (**F**).

The values of waist circumference in the diabetic female population were positively and significantly correlated with TNF-α (r=0.340, p=0.040), VEGF (r=0.414, p=0.011), hsCRP (r=0.448, p=0.022) levels, while in the male diabetic population none of these correlations were statistically significant and the associations of WC with VEGF and with hsCRP showed an inverse profile (r=0.264, p=0.105; r=−0.227, p=0.164 and r=−0.222, p=0.408, respectively) (Figure 3A, 3B and 3C). However, waist circumference was positively and significantly correlated with uric acid in males (r=0.339, p=0.035) but not in females (r=0.250, p=0.261) (Figure 3D). Furthermore, also in the female diabetic subjects, serum TNF-α concentrations showed a positive and significant correlation with VEGF (r=0.282, p=0.044) levels and hsCRP contents, which presented an inverse and significant correlation with adiponectin (r=−0.590, p=0.004) concentrations, which were less evident and statistically non-significant in the male diabetic patients (r=−0.027, p=0.853; r=−0.164, p=0.490) (Figure 3E and 3F).

**Figure 4:**
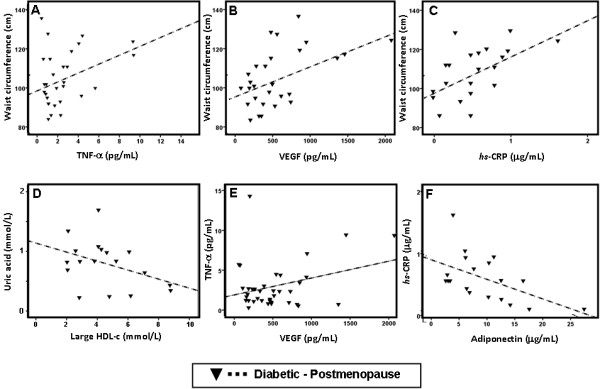
**Main correlations in postmenopausal diabetic patients.** Correlation between waist circumference and TNF-α (**A**), VEGF (**B**) and *hsCRP* (**C**); between uric acid and large HDL-c (**D**); between TNF-α and VEGF (**E**) and between *hsCRP* and adiponectin (**F**).

Regarding the postmenopausal diabetic population, once again, there was a strong positive and significant correlation between WC and TNF-α (r=0.354, p=0.046), WC and VEGF (r=0.446, p=0.014) and WC and hsCRP (r=0.496, p=0.019) levels (Figure 4A, 4B and 4B). Serum uric acid presented an inverse and significant correlation with large HDL-c (r=−0.405, p=0.045) (Figure 4D). Additionally, in the same population of postmenopausal diabetic patients, serum TNF-α concentrations showed a positive and significant correlation with VEGF (r=0.302, p=0.040) levels and hsCRP contents presented an inverse and significant correlation with adiponectin (r=−0.534, p=0.018) concentrations (Figure 4E and 4F). No correlation analysis was performed for premenopausal diabetic patients due to the short number of individuals in this subpopulation, which makes unfeasible this type of analysis.

## Discussion

The risk for coronary artery disease (CAD), the main cause of death in women, increases after menopause [9]. Premenopausal women are at lower risk for CAD than postmenopausal and than men [8,9]. Although previous studies have spotlighted the effects of estrogens, no conclusive evidence has proven their role in reducing the incidence of CVD [27]. Indeed, hormone replacement therapy for the menopausal women does not confer cardiovascular protection according to the Women’s Health Initiative trial [28]. Therefore, estrogen deficiency may indirectly contribute to the increased risk of CVD in postmenopausal women. Some studies suggest that the cardiovascular effects usually attributed to menopause are merely a consequence of the older age of menopausal women [29]. In contrast, others demonstrated that menopause is associated with a modest increase in total fatness and an accelerated accumulation of central body fat that exceeds changes normally attributed to the aging process [30]. Indeed, the transition from premenopausal to postmenopausal status is associated with the emergence of various risk factors for metabolic syndrome and the rising incidence of CAD during menopause occurs in parallel with an increase in the incidence of T2DM [10]. The presence of diabetes increases the risk for CAD in both premenopausal and postmenopausal women and probably counteracts the protective effect of estrogens on the vasculature [31,32], so that premenopausal diabetic women show the same risk for CAD as men and 2- to 5-fold higher rates than in non-diabetic women [12,13]. In addition, CAD is considered as one of the most important complications of DM in both sexes. Hypertension and dyslipidemia are risk factors for CAD among diabetic patients and it is well established that patients with diabetes have more extensive and more rapidly progressive CAD than non-diabetic subjects [12,13]. Several studies showed a 2 to 4-fold higher prevalence of atherosclerotic disease in diabetic compared to non-diabetic individuals [33,34]. Diabetic women have greater mortality risk from CAD than non-diabetic men and women [35]. Since the traditional CVRFs cannot completely account for these sex differences in cardiovascular mortality, more research is pivotal to understand the precise influence of gender and menopause in the risk for CVD, especially in diabetic patients.

This study has compared the effects of gender and menopause on cardiovascular parameters/markers in a diabetic population under antidiabetic, antidyslipidemic and antihypertensive medication, compared with matched controls. Male and female subpopulations, from both groups, presented identical age, percentage of males/females and BMI. Female waist circumference normal range is distinct between males and females cannot be directly compared. As expected, diabetic patients (from both genders) presented higher values of glycemia, BMI and WC, when compared with the age and gender-matched controls. However, systolic and diastolic blood pressure were significantly reduced in the diabetic patients (male and female), confirmative of the antihypertensive medication taken. The data of the control subjects shows slight higher values of blood pressure that might be related with the age of these individuals, which were chosen to be age-matched with the diabetic ones in order to minimize the influence of this factor in the analysis. Additionally, lower values of total-c, LDL-c, Ox-LDL-c and non-HDL-c were found in the diabetic patients when compared with the control subjects. However, increased contents of serum TGs and reduced of HDL-c were found for the diabetic subjects. This classical lipid profile is in agreement with what could be expected with the type of antidyslipidemic therapy practiced, since statins, the main class of drugs used, have less impact on TGs and HDL-c than on total-cholesterol and LDL-c. Despite the pharmacological and behavioral interventions directed to control risk factors for CVD in diabetic patients, the incidence of CVD remains alarmingly high. Dyslipidaemia, which is associated with increased CVD mortality in diabetic individuals [36], is among the most important modifiable risk factors. There is now a wide variety of antidyslipidemic drugs [37,38]; however, with the increasing combination of risk factors commonly found in several CV diseases, including in T2DM, the control of dyslipidaemia is not enough, requiring a more effective modulation of HDL-c, which remains only slightly modifiable with the current pharmacological arsenal [39,40]. Our study in diabetic patients under antidyslipidemic therapy reinforces the need of better intervention on HDL-c.

Concerning other markers of cardiovascular/metabolic risk, the male diabetic patients presented increased hsCRP, uric acid and reduced adiponectin concentrations, while the female diabetic subjects also presented increased hsCRP and uric acid, as well as TNF-α and VEGF, with reduced adiponectin levels. Thus, both the male and female diabetic patients showed impaired markers of cardiometabolic risk, which is accompanied by increased waist circumference. This pro-inflammatory profile, a key factor in the development of atherosclerosis, is in agreement with other studies which have reported increased pro-inflammatory mediators, such as TNF-α and hsCRP, and reduced levels of anti-inflammatory and anti-atherogenic mediators, such as adiponectin [21,41]. Furthermore, adiponectin levels of diabetic woman were negatively associated with hsCRP, in agreement with a recent study [42]. Additionally, increase in hsCRP levels was greater in women than men, in agreement with previous data [43], and might be due to the significantly increased concentration in the premenopausal diabetic women. Hence, even though the reduced blood pressure and some of the traditional marker of lipid profile (total-c, LDL-c and non-HDL-c), which is in agreement with the medication taken, the diabetic patients presented obesity and visceral adiposity, accompanied by markers of low-grade inflammation, and uncorrected TGs and HDL-c contents, which are less modifiable with the most used antidyslipidemic agents (mainly statins). Collectively, the impaired parameters might be viewed as predictors/markers of an increased cardiometabolic risk in this diabetic population.

Regarding the differences among men and women, in the control population females presented lower glycemia, unchanged total-c, TGs, LDL-c and non-HDL-c, together with reduced Ox-LDL, TNF-α, adiponectin and uric acid. However, lower PON1 activity was found in women, which might be explained by the men’s compensatory increment of PON1 activity against the pro-oxidative profile (viewed by the significantly higher Ox-LDL contents). In agreement, despite identical total HDL-c concentrations in both subgroups, the quality of HDL was better in female, as they presented higher contents of large HDL-c and reduced of small HDL-c. Therefore, collectively, this data is indicative of a better cardiometabolic profile and lower CV risk in non-diabetic females when compared with males. However, when the subpopulations of diabetic patients are compared, the differences between male and female are significantly reduced. Indeed, almost all the parameter of reduced risk found in the control females (vs control males) are no long different, and women even presented higher values of HbA1c, VEGF and hsCRP, despite the higher adiponectin concentration, which might be viewed as the exception of this clearly worse cardiometabolic profile. If compared with the female control population, this indication is even clearer. The differences of adiponectin encountered might be due to differences on fat distribution (which is known to differ between genders, with men having more visceral and less subcutaneous fat) as well as due to the effect of sex hormones, that are involved in the metabolism of adipose tissue and fat distribution. Our results may be due to increased visceral fat in female control population (proportionately larger than the male). However, when comparing the diabetic female population with control female we found that adiponectin levels are decreased in diabetic women, in agreement with previous data [44], which is consistent with the other results of our study that show a worse cardiometabolic profile of women in the presence of T2DM. The significant decrease of adiponectin levels in diabetic men, when compared with control men, is consistent with other studies which have previously suggested that low adiponectin levels are associated with fatty liver disease in women and low testosterone levels in men with type 2 diabetes [45].

Diabetic women presented not only the expected increased glycemia and obesity (higher BMI and WC) when compared with the female control subjects, but also increased hsCRP, TNF-α, uric acid and VEGF, accompanied by reduced adiponectin. Additionally, despite unchanged values of classical lipid profile (total-c, LDL-c, non-HDL-c), due to medication, the diabetic females showed increased TGs and reduced HDL-c quality, confirmed by the reduced amount of large HDL-c and increased of small HDL-c, regardless of normal total HDL-c quantity. Low levels of HDL-c are a major CHD risk factor in type 2 diabetic subjects [46]. In T2DM patients, TGs are usually elevated and HDL metabolism is perturbed with evidence of both qualitative and quantitative alterations [17]. Despite the growing body of evidence indicating that determination of HDL subpopulations may add an important information on CHD risk prediction [15,18], data on HDL subpopulation distribution and its modulating factors in women when compared with male are limited, including in diabetic populations. Our data is in agreement with the study of Russo et al. (2010) that showed a lipid and HDL subpopulation profiles more atherogenic in the diabetic women [47].

Some of the markers of cardiometabolic risk analyzed showed important correlations in the female diabetic population, in opposition with the data obtained in the male one, reinforcing the previous data and indications. Indeed, the waist circumference in the diabetic women is positively and significantly correlated with the concentrations of hsCRP, TNF-α and VEGF, which was not encountered in the male diabetic individuals, for whom the increased uric acid is indeed the best association with waist circumference. Additionally, TNF-α contents are positively and significantly correlated with VEGF levels and hsCRP concentrations are inversely and significantly correlated with adiponectin values. Collectively, this data suggests that obesity, and especially abdominal adiposity, in female diabetic subjects, is metabolically more active and, consequently, deleterious than in male. This data is in agreement with the fact that women with T2DM, compared with age-matched non-diabetic women, exhibit several-fold higher rates of death related to CAD, with event rates nearly identical to those observed in T2DM men [13], as is also in line with other study in type 1 diabetes [48]. Our data suggested that diabetes abrogate the protective effect of gender encountered in women without diabetes, which agrees with the suggestion that CHD risk is higher in men and that the difference is reduced in diabetic populations [49]. Nevertheless, the traditional CVRFs fail to completely explain these sex differences, and the new “non-classical” factors seem to be able to improve knowledge and clarify this discrepancies, in particular the more atherogenic lipid sketch and pro-inflammatory and pro-angiogenic profile, viewed mainly by the contents of HDL-c subpopulations and the serum values of TNF-α, hsCRP and VEGF.

Regarding the influence of menopause, glycemia was significantly higher after menopause, both for control and diabetic females. However, postmenopausal diabetic woman presented significantly increased BMI and waist circumference when compared with the control postmenopausal subjects, while no changes were observed between groups at premenopausal stage. Systolic blood pressure was significantly increased after menopause, for both control and diabetic groups. However, diabetic patients presented a trend to reduced SBP and significantly lower values of DBP, when compared with the control population, both for pre and postmenopause comparisons, which might be due to antihypertensive medication. In relation to the classical lipid profile, in both the 2control and diabetic groups, no changes were encountered between pre and postmenopausal women for total-c, TGs, LDL-c and non-HDL-c. Additionally, both pre and postmenopausal diabetic patients presented unchanged or even decrease (LDL-c) values for all of those parameters. This profile is in agreement, again, with the type of antidyslipidemic therapy practiced by those patients, mainly statins, which decrease total-c and LDL-c contents, but have less impact on TGs and HDL-c levels. Indeed, TGs concentrations were significantly higher, but only in the postmenopausal diabetic women when compared with the control postmenopausal subjects. In addition, although total HDL-c concentration was unchanged in pre and postmenopausal diabetic women, when compared with the control matched subpopulations, in the postmenopausal stage the HDL-c quality is worse, viewed by the significantly reduced proportion of large HDL-c and increased of small HDL-c. Although further studies are needed to clarify the complex role of the different HDL particles in the development of CHD, several lines of evidence indicate that evaluating HDL subpopulations profile may provide some adjunctive information on CHD risk definition, independently from total HDL-c measurements [15,18]. Our data clearly reinforces this recommendation, in particular in the postmenopausal diabetic women, in whom the quality of HDL is poor.

Concerning other markers of cardiovascular/metabolic risk, before menopause the diabetic women presented significantly increased hsCRP, uric acid and VEGF, with a trend to reduced adiponectin contents, when compared with the non-diabetic females. These changes were maintained after the menopause, accompanied by increased TNF-α concentration. In this population, an important association was found between some of the markers of cardiometabolic risk. Indeed, the waist circumference in the postmenopausal diabetic women is positively and significantly correlated with the concentrations of hsCRP, TNF-α and VEGF. Additionally, TNF-α contents are positively and significantly correlated with VEGF levels, hsCRP concentrations are inversely and significantly correlated with adiponectin values and, finally, uric acid was inversely and significantly correlated with large HDL-c. Thus, the postmenopausal diabetic females have an increased obesity which seems to be metabolically more deleterious than in premenopausal diabetic women, namely due to obesity-induced low-degree chronic inflammation, through enhanced adipose tissue-derived cytokine expression, in agreement with previous recent reports [50]. The transition from pre to postmenopause may be associated with features of the metabolic syndrome, including an increased central body fat, a shift toward a more atherogenic lipid profile, as well as other risk factors. Obesity is an independent risk factor for macrovascular disease across sexes, but despite higher incidence of obesity in premenopausal women, rates of macrovascular disease are lower in premenopausal women than in men. Interestingly, this sex difference, which normally vanishes after menopause, is rapidly lost in premenopausal DM patients, with CVD reaching 2- to 5-fold higher rates than in non-diabetic women [12]. In fact, women with T2DM, compared with age-matched non-diabetic women, exhibit significantly higher rates of death related to CAD, similar to those observed in T2DM men [13]. The emergence of the CVD risk factors in the postmenopausal women may be a direct result of ovarian failure, or an indirect result of the metabolic consequences of body fat centralization with estrogen deficiency. Previous studies have also demonstrated that menopause is associated with a modest increase in total fatness and an accelerated accumulation of central body fat that exceeds changes normally attributed to the aging process [30,51]. Similarly in our studies, postmenopausal women, when compared to premenopausal women, had a higher WC, TGs level and other non classical markers, suggesting an increased CV risk. Previous reports have indicated that the diabetic postmenopausal women have more severe CAD and CV risk compared to non-diabetic women [52].

Although the small number of premenopausal diabetic women could be viewed as a study limitation, that deserves further strengthening, postmenopausal diabetic women have a clearly poor cardiometabolic profile since several parameters that are unchanged between premenopausal diabetic subjects when compared with the premenopausal controls, are aggravated in the postmenopausal diabetic ones, versus the corresponding postmenopausal controls, including significantly increased TGs, small HDL, WC, BMI and TNF-α values, aggravated contents of VEGF and uric acid, significantly lower of large HDL and a trend to reduced adiponectin concentration. Collectively, our data reinforces the suggestion that the multi-targeted treatment of all risk factors is even more justified in postmenopausal women, which is mainly suggested by non-traditional markers in this diabetic population medicated for hypertension and dyslipidemia. The apparently more deleterious visceral obesity, the more atherogenic lipid sketch and the pro-inflammatory profile in diabetic patients in general, but in the postmenopausal women in particular, urges precise attention and proper multi-therapeutic intervention.

## Conclusions

Our study suggests that diabetes abrogate the protective effect of female gender on non-diabetic subjects when compared with male, and that postmenopausal diabetic females presented worsen cardiometabolic profile, including a more atherogenic lipid sketch and a pro-inflammatory and pro-angiogenic profile. The traditional CVRFs fail to completely explain these differences, which are better clarified using “non-classical” markers, such as contents of HDL-c subpopulations, rather than total content, and mediators of inflammation and angiogenesis, namely TNF-α, hsCRP, uric acid and VEGF. Multi-therapeutic intervention, directed to obesity, atherogenic lipid particles and inflammatory mediators, is advisory in order to efficiently prevent the serious cardiovascular complications of diabetes in this higher-risk population.

## Abbreviations

BMI: Body mass index; CAD: Coronary artery disease; CHD: Coronary heart disease; CV: Cardiovascular; CVD: Cardiovascular disease; CVR: Cardiovascular risk; CVRFs: Cardiovascular risk factors; DBP: Diastolic blood pressure; DM: Diabetes mellitus; HbA1c: Glycated hemoglobin; HDL-c: High-density lipoprotein cholesterol; hsCRP: High sensitivity C reactive protein; iCAM1: Intercellular adhesion molecule 1; LDL-c: Low-density lipoprotein cholesterol; OAD: Antidiabetics; Ox-LDL-c: Oxidized low-density lipoprotein cholesterol; PON1: Paraoxonase 1; SBP: Systolic blood pressure; T2DM: Type 2 diabetes mellitus; TGs: Triglycerides; TNF-α: Tumor necrosis factor alpha; Total-c: Total cholesterol; VEGF: Vascular endothelial growth factor; WC: Waist circumference

## Competing interests

The authors declare that they have no competing interests.

## Authors’ contributions

FMM, FT and FR conceived and designed the study protocol. FMM, DM, FP, JS, AC, RP and ETL provided collection of data and performed analyses. FMM, FT and FR prepared the manuscript. All authors have read and approved the manuscript.
